# Prolonged Graft Survival in Older Recipient Mice Is Determined by Impaired Effector T-Cell but Intact Regulatory T-Cell Responses

**DOI:** 10.1371/journal.pone.0009232

**Published:** 2010-02-16

**Authors:** Christian Denecke, Damanpreet Singh Bedi, Xupeng Ge, Irene Kyung-eun Kim, Anke Jurisch, Anne Weiland, Antje Habicht, Xian C. Li, Stefan G. Tullius

**Affiliations:** 1 Division of Transplant Surgery and Transplant Surgery Research Laboratory, Brigham and Women's Hospital, Harvard Medical School, Boston, Massachusetts, United States of America; 2 Transplantation Research Center, Brigham and Women's Hospital and Children's Hospital of Boston, Boston, Massachusetts, United States of America; 3 Transplant Research Center, Beth Israel Deaconess Medical Center, Harvard Medical School, Boston, Massachusetts, United States of America; New York University, United States of America

## Abstract

Elderly organ transplant recipients represent a fast growing segment of patients on the waiting list. We examined age-dependent CD4^+^ T-cell functions in a wild-type (WT) and a transgenic mouse transplant model and analyzed the suppressive function of old regulatory T-cells. We found that splenocytes of naïve old B6 mice contained significantly higher frequencies of T-cells with an effector/memory phenotype (CD4^+^CD44^high^CD62L^low^). However, in-vitro proliferation (MLR) and IFNγ-production (ELISPOT) were markedly reduced with increasing age. Likewise, skin graft rejection was significantly delayed in older recipients and fewer graft infiltrating CD4^+^T-cells were observed. Old CD4^+^ T-cells demonstrated a significant impaired responsiveness as indicated by diminished proliferation and activation. In contrast, old alloantigen-specific CD4^+^CD25^+^FoxP3^+^ T-cells demonstrated a dose-dependent well-preserved suppressor function. Next, we examined characteristics of 18-month old alloreactive T-cells in a transgenic adoptive transfer model. Adoptively transferred old T-cells proliferated significantly less in response to antigen. Skin graft rejection was significantly delayed in older recipients, and graft infiltrating cells were reduced. In summary, advanced recipient age was associated with delayed acute rejection and impaired CD4^+^ T-cell function and proliferation while CD4^+^CD25^+^FoxP3^+^ T-cells (Tregs) showed a well-preserved function.

## Introduction

In clinical transplantation, advanced recipient age has been described as an independent risk factor for long-term allograft deterioration [1]. On the other hand, reports on the influence of age on acute rejection remain controversial [2]–[4]. Besides, experimental studies on the association of recipient age and graft outcome are limited. Pascher et al. reported an enhanced immune responsiveness of elderly recipients early after transplantation [5] while Sun et al. found no differences in skin graft survival despite an impaired in vitro response to alloantigen [6].

A decline in CD4^+^ T-cell function with increasing age has been well described. Naive aged CD4^+^ T-cells were shown to have an impaired response to alloantigen, as shown by reduced IL-2 production, less proliferation upon activation, and impaired effector T-cell generation [7]. In addition, memory T-cells generated from naive aged T-cells were shown to be hyporesponsive and exhibited poor helper function [8]. Previously, Rosenberg et al. reported on the delayed rejection of MHC class II disparate skin grafts in old recipients, suggesting an impaired function of accumulating memory CD4^+^ T-cells [9].

Recently, mechanisms of T-cell mediated graft rejection in old recipients have been examined by Tesar et al. who demonstrated that aged skin graft recipients exhibit an impaired memory T-cell response that is associated with impaired Th1 mechanisms and elevated IL-17 production [10]. We sought to examine the influence of age-dependent changes of CD4^+^T-cell function on skin graft rejection. A recently described adoptive transfer model allowed us to specifically investigate activation and migration of alloantigen-specific CD4^+^ T-cells [11], [12]. As effector and regulatory mechanisms may balance the immune response differently in young and old animals, we also examined the functional properties of young and old CD4^+^CD25^+^FoxP3^+^T-cells. To our knowledge, our experiments resemble the first study examining the role of aged effector CD4^+^ T-cells and regulatory T-cells in the setting of organ transplantation.

## Materials and Methods

### Mice

3mths old C57BL/6, B6.C-H2^bm12^ (bm12: weight:18 g–25 g) and C57BL/6 nude mice mice were purchased from The Jackson Laboratory (Bar Harbor, ME). 18mths old C57BL/6 mice were obtained from the National Institute of Aging (NIA, Bethesda, MD, weight: 40±5g). ABM TCR-Tg mice were a kind gift from Dr. Sayegh and bred in our animal facility in accordance with Institutional Guidelines. ABM mice were used at 3mths or at 15 to 18mths of age.

### Skin Transplantation

Full thickness bm12 skin grafts were engrafted onto the dorsolateral thorax of recipient mice using 5–0 Vicryl, covered with gauze and a securing bandage for one week. Skin graft survival was monitored daily and rejection was defined as graft necrosis of more than 80%. Graft survival was independently assessed by investigators blinded for the particular experimental groups.

Bilateral skin grafts were used in all experiments in order to optimize the yield of alloantigen-specific transgenic cells from draining lymph nodes (dln).

### T-cell Purification and Adoptive Transfer

Adoptive transfer experiments were performed as previously described (11). Briefly, CD4^+^ T-cells were isolated by positive selection using magnetic beads associated cell sorting (MACS) from both spleens and lymph nodes of ABM transgenic mice (Miltenyi Biotec, Auburn,CA). CD4^+^ T-cells were passed twice through MACS columns in order to increase purity to >90%. A sample of cells was stained with anti-CD4, anti-TCR Vα2.1 and anti-TCR Vβ8.1 and analyzed to determine the percentage of TCR-tg CD4^+^ T-cells. More than 90% of isolated CD4^+^ T-cells expressed the transgenic TCR.

2×10^6^ naïve young or old CD4^+^ Vα2.1^+^ Vβ8.1^+^ T-cells were intravenously injected into B6 nude recipients one day prior to bm12 skin transplantation. On day 7 after transplantation, ABM transgenic T-cells were harvested from spleens, draining lymph nodes (axillary) and non-draining lymph nodes (pooled cervical, inguinal, mesenteric) and analyzed via flow cytometry in single cell suspensions.

### Flow Cytometry

Cells were washed in PBS containing 2% Rat-Serum (Bio Whittaker, Walkersville, MD) to block unspecific FcR-binding. In WT mice, lymphocytes were stained with directly conjugated anti-CD4, anti-CD69, anti-CD25, anti-CD44, anti-CD62L or chemokine receptor antibodies. For FoxP3-staining, a commercially available kit (eBioscience) was used.

In transgenic experiments, 1×10^6^ cells were stained with anti-CD4 (PerCP), anti-TCR Vα2.1 (FITC) and anti-TCR Vβ8.1 (Strepavidin-APC) mAbs to identify TCR tg T-cells. Subsequently, cells were stained with PE-conjugated anti-CD25, anti-CD69 (activation markers), anti-CXCR3, anti-CCR5, or anti-CCR7 mAbs. For detection of CD4^+^ T-cells with a memory/effector phenotype, cells were stained with anti-CD4 (PerCP), anti-CD44 (APC) and anti-CD62L (FITC).

All mAbs were purchased from BD Biosciences (San Diego, CA).

### Intracellular Cytokine Staining

1×10^6^ cells were resuspended in cell media (HL-1 media containing 10%FCS, 1% L-Glutamine, 1% Penicillin/Streptomycin, all Bio Whittaker, Walkersville, MD) and re-stimulated with PMA (5 ng/ml) and Ionomycin (500 ng/ml) (Sigma, St. Louis, MO). Brefeldin A (10 µg/ml) (Sigma, St. Louis, MO) was added as Golgi-Stop; cells were incubated for 4 hrs at 37°C. Unstimulated cells served as controls. Following stimulation and surface staining (CD4, Vα2.1 and Vβ8.1), cells were permeabilized (Cytofix/Cytoperm, BD Biosciences, San Diego, CA) and stained with PE-conjugated anti-IFNγ, IL-2, IL-4 or IL-10 mAbs and isotype control mAbs.

### Suppression Assay

1×10^5^ CD4^+^CD25^-^ T-cells and CD4^+^CD25^+^ Tregs were isolated from allo-sensitized young (3mths) and old BL/6 mice (18mths) and, at varying ratios, co-cultured for 72 hrs in the presence of irradiated bm12 stimulator cells. Following 12 hrs of incubation with ^3^H TdR (Thymidine) the proliferation of responder cells was determined by thymidine incorporation.

### ELISPOT Assay

Splenocytes from naive or transplanted B6 mice were harvested and subsequently stimulated with naive wild-type bm12 splenocytes. The ELISPOT assay was employed to measure the frequency of alloreactive T-cells producing IFNγ (Th1) as previously described [5] . The resulting spots were counted on a computer-assisted enzyme-linked immunospot image analyzer (Cellular Technology), and frequencies were expressed as the number of cytokine-producing spots per 0.5×10^6^ splenocytes.

### Proliferation Assay (Mixed Lymphocyte Culture)

B6 splenocytes were isolated and 0.5×10^6^ cells/well were co-cultured with 0.5×10^6^ bm12 splenocytes in round-bottomed 96-well plates (BD Biosciences). After 72 hrs, plates were pulsed for 12hrs with 1 µCi [^3^H]thymidine per well. Proliferation was measured as counts per minute by using a Wallac Liquid Scintillation Counter (Perkin Elmer).

### Immunohistochemistry

Immunohistochemistry was performed using OCT-embedded, 4um thick acetone fixed mouse tissue sections. Slides were pre-treated with Peroxidase Block (DAKO USA, Carpinteria, CA) for 5 minutes to quench endogenous peroxidase activity. For CD4, monoclonal rat anti-murine CD4 (clone 4B12, Vector Labs, Burlingame, CA, Cat# VP-C319) was applied in DAKO diluent at 1∶200 for 1 hour, washed, and then rabbit anti-rat immunoglobulin antibody was applied at 1∶750 in DAKO for 1 hour. Slides were washed in 50-mM Tris-Cl, pH 7.4, and detected with anti-rabbit Envision+ kit (DAKO) as per manufacturer's instructions. After further washing, immunoperoxidase staining was developed using a DAB chromogen (DAKO) and counterstained with hematoxylin.

CD4^+^ T-cells in 5 high power fields were counted and stated as mean ± SD.

### Statistics

Comparisons between experimental groups were performed using Student's t test. All results were generated using GraphPad Prism software (San Diego, CA). A p-value<0.05 was considered statistically significant.

## Results

### Age-Dependent Alterations of T-Cell Subsets, Cytokine Production and Proliferation

**Figure 1:**
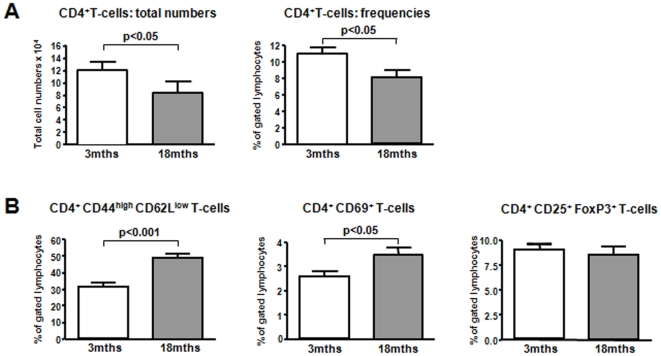
Altered T-cell repertoire in aged naive mice. Contraction of the T-cell repertoire in aged naive mice: 3 and 18mths naive B6 mice were sacrificed and splenocytes stained for CD4^+^T-cell subpopulations (n = 6). CD4^+^T-cells were reduced in frequency and total numbers in older animals (**A**). Effector/memory CD4^+^T-cells and early activated CD4^+^T-cells accumulated in older animals while frequencies of regulatory T-cells were age-independent (**B**).

In order to examine the T-cell response in old animals, we first compared splenocytes phenotypically in naïve 3mths and 18mths old B6 mice. CD4^+^ T-cells were grossly reduced both in frequencies and absolute numbers in aged spleens. At the same time we found significantly higher frequencies of early-activated CD4^+^CD69^+^ T-cells and CD4^+^ effector/memory T-cells (CD44^high^CD62L^low^) in old animals (**Fig. 1**). Interestingly, frequencies of CD4^+^CD25^+^FoxP3^+^ regulatory T-cells were comparable in old and young animals.

**Figure 2:**
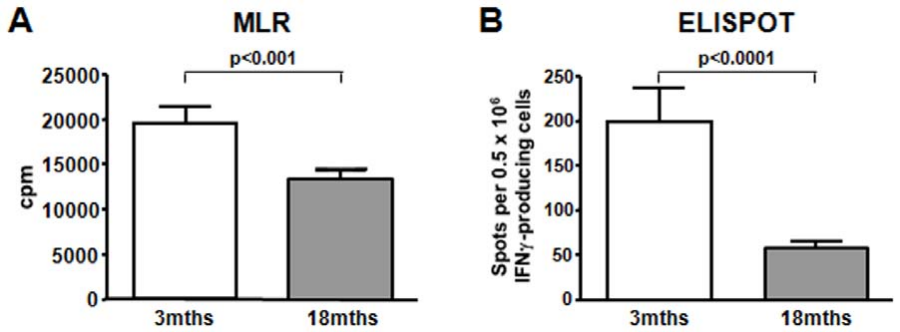
Naive old mice demonstrate a poor functional response to antigen in vitro. Naive old mice demonstrate a poor functional response to allo-antigen in vitro; (**A**) Splenocytes from naive B6 mice were co-cultured with bm12 antigen for 72hrs. Following 12hrs of incubation with 3H TdR (Thymidine) the proliferation of responder cells was determined by Thymidine incorporation. (**B**) Young and old B6 splenocytes were co-cultured in ELISPOT plates and resulting IFNγ spots counted. Both, T-cell proliferation (**A**) and alloreactive IFNγ -production (**B**) were significantly impaired in aged mice indicating a reduced functional T-cell response (n = 6).

Next, we stimulated young and old WT B6 splenocytes in vitro with bm12 antigen in order to examine proliferation and cytokine production. Old splenocytes demonstrated a significantly reduced proliferation in mixed lymphocyte reaction (MLR). Old splenocytes also exhibited a significantly impaired IFNγ - production as shown by ELISPOT (**Fig. 2**).

Taken together we observed a higher frequency of effector/memory T-cells but an impaired in vitro proliferation and IFNγ production in old mice. Interestingly, the frequency at which regulatory T-cells were found remained unaltered between the two groups.

### Skin Graft Rejection in WT Mice

**Figure 3:**
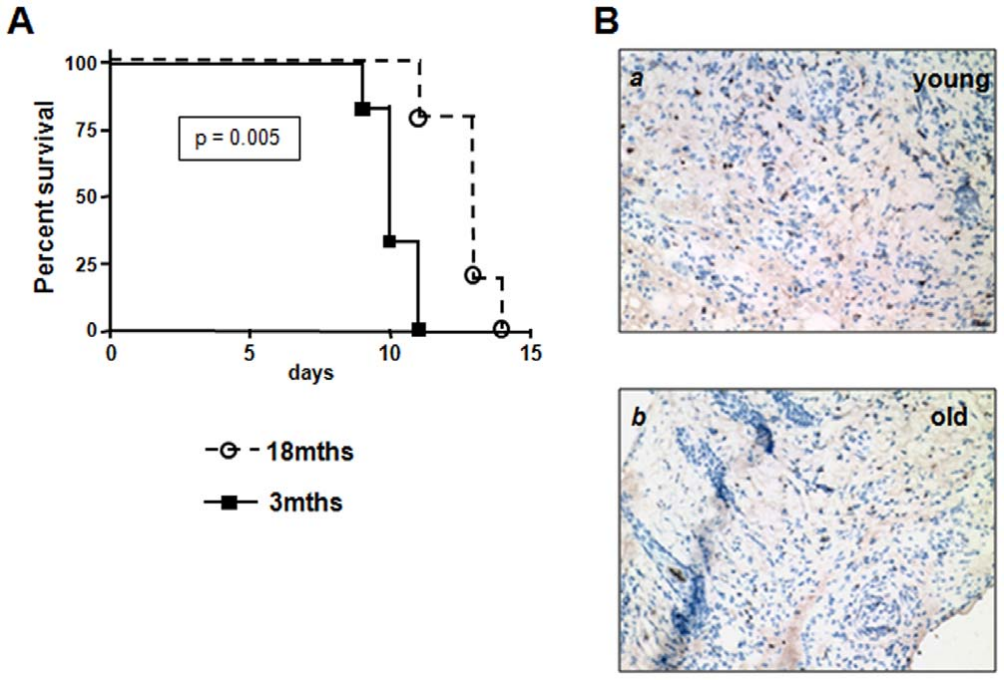
Recipient age prolongs skin allograft survival in WT mice. Recipient age prolongs skin allograft survival in WT mice (**A**), (p<0.005) and is associated with reduced intragraft CD4^+^ T-cell infiltrates (**B**) (day 7, 200×).

**Figure 4:**
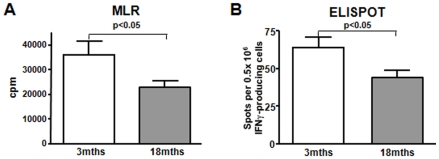
Impaired immune response in old WT recipients. Old WT animals demonstrate an impaired immune response after transplantation: Both, T-cell proliferation (**A**) and alloreactive IFNγ -production (**B**) were significantly reduced in aged mice indicating a poor T-cell response upon re-stimulation in vivo (n = 6).

Next, we examined the effects of the aging immune system on the recipient's alloimmune response by engrafting 18mths B6 mice with bm12 skin transplants. In keeping with the poor response to antigen seen in our in vitro analyses of aging mice, old recipients also showed a significantly delayed graft rejection compared to young recipients (13 vs. 10 days, p = 0.005). However, CD4^+^ T-cell infiltrates were comparable by day 7 (**Fig. 3**). As in our findings in naïve old mice, proliferation of splenocytes harvested from aged skin graft recipients was significantly reduced in MLR after 7 days. Likewise, alloreactive IFNγ production, as assessed by ELISPOT was also impaired (**Fig. 4**). Next, T-cell activation and differentiation was analyzed in spleens and draining lymph nodes (dln) in young and old skin graft recipients early after transplantation.

### Early T-Cell Activation, Cytokine and Chemokine Expression

**Figure 5:**
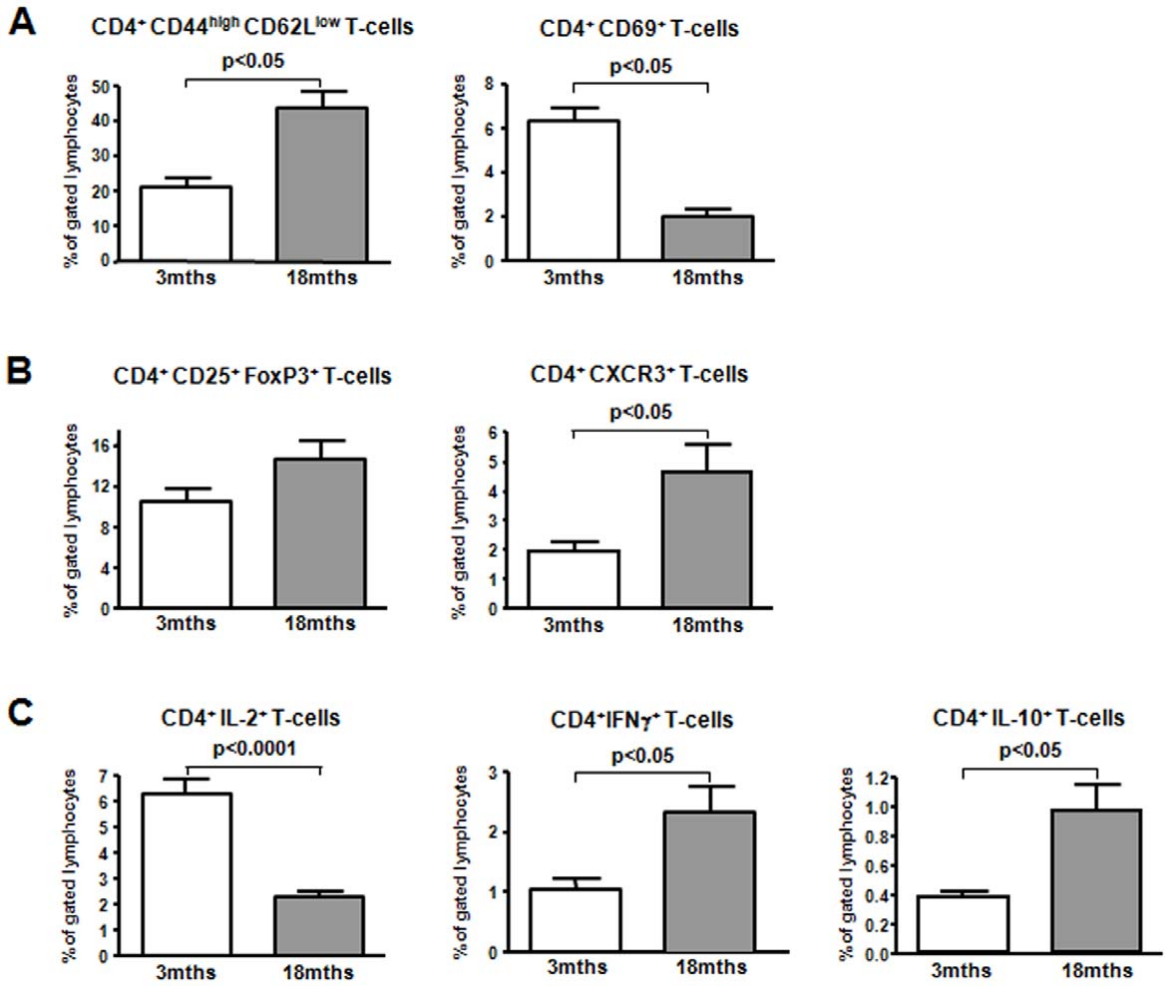
Impaired T-cell activation and proliferation in aged WT recipients. Impaired T-cell activation and proliferation of splenocytes of aged WT transplant recipients. 3 and 18mths old WT recipients of bilateral bm12 skin grafts were sacrificed by day 7 and splenocytes were re-stimulated with PMA/Ionomycin for 4hrs. Following surface staining (CD4, Vα2.1 and Vβ8.1) cells were permeabilized and stained with PE-conjugated anti-IFNγ, IL-2 or IL-10 mAbs and isotype control mAbs. Early activated CD4^+^T-cells were reduced in frequency in older animals (**A**) while effector/memory CD4^+^T-cells and regulatory CD4^+^T-cells accumulated. (**B**) Despite a reduced functional in vitro and in vivo response as indicated by impaired IL-2 production expression of proinflammatory cytokines is elevated with age at day 7 after restimulation (**C**).

In accordance with the overall reduced proliferation of old T-cells, FACS analysis of splenocytes from old recipients of skin grafts showed a significantly lower percentage of CD4^+^IL-2^+^ T-cells and early-activated CD4^+^CD69^+^ T-cells. While higher percentages of CD4^+^ effector/memory T-cells were found in old mice, the majority of T-cells were hyporesponsive as indicated by prolonged graft survival and compromised function in MLR and ELISPOT analysis. Interestingly, intracellular FACS staining revealed higher frequencies of IFNγ^+^ T-cells in old mice, most likely attributable to the expanded effector/memory CD4^+^ T-cell population (**Fig. 5**). However, when these memory T-cells were re-stimulated in vitro with alloantigen (ELISPOT), IFNγ release was significantly impaired underscoring previous findings of an impaired Th1 cytokine release upon activation by aged memory T-cells [8]. Likewise, recent studies demonstrated an impaired cytokine production after viral infection in older animals [13].

While no differences in regulatory T-cells were seen in naive young versus old mice prior to transplantation, 7 days after skin engraftment significantly higher frequencies of CD4^+^CD25^+^FoxP3^+^ regulatory T-cells were detected in draining lymph nodes of old mice (data not shown). These results indicate that the regulatory response may be well preserved with increasing age. In order to examine migratory capacities of young and old T-cells, expression of different chemokine receptors such as CXCR3, CCR5 and CCR7 that are crucial for allograft rejection were analyzed [14]–[16]. Our experiments demonstrated a significantly delayed graft rejection of aged recipients associated with a diminished in vitro response after transplantation. Despite accumulation of effector/memory CD4^+^ T-cells the majority of T-cells remained hyporesponsive as indicated by decreased expression of activation/proliferation markers such as CD25, CD69, and IL-2. While homing characteristics of old T-cells seem to be unchanged after transplantation, significantly higher frequencies of regulatory T-cells were found in draining lymph nodes of old recipients. Thus, we sought to further investigate the role of an expanded regulatory T-cell population and it's impact on a diminished alloimmune response in aged recipients.

### Regulatory T-Cell Function Remains Intact with Age

**Figure 6:**
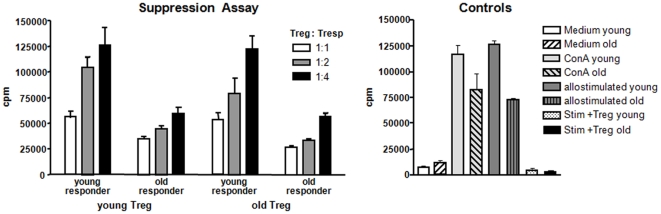
Regulatory T-cell function remains unaltered with age. Regulatory T-cell function remains age-independent: CD4^+^CD25^−^ T-cells (responder cells) and regulatory T-cells (FoxP3^+^) were co-cultured at different ratios in the presence of stimulator cells. The proliferation of responder cells was determined by Thymidine incorporation. Young and old Tregs suppressed the proliferation of responder cells in a dose-dependent manner. Co-culture of old Tregs with young responders cells demonstrated a well-preserved suppressor function of old regulatory T-cells comparable to the suppressor function of young regulatory T-cells.

As the alloimmune response in transplantation resembles a balance between effector and regulatory mechanisms, we sought to examine the in vitro function of allospecific young and old CD4^+^CD25^+^FoxP3^+^ T-cells. Young regulatory T-cells suppressed the proliferation response of allospecific young responder T-cells in a dose-dependent manner (**Fig. 6**). Old regulatory T-cells showed a similar suppressor function compared to young T-regs when co-cultured with young responder cells.

These findings indicate a well-preserved suppressive function of old allospecific regulatory T-cells thereby contributing to the delayed rejection kinetics seen in old animals.

### Skin Graft Rejection after Adoptive Transfer (AT) of Young and Old Allospecific tg TCR^+^CD4^+^T-Cells

In order to further examine our findings of reduced T-cell responsiveness and to elucidate the age-dependent effects of alloantigen-specific T-cells, a recently developed adoptive transfer model was used [12]. Specifically, anti-bm12 specific CD4^+^ T-cells from ABM transgenic mice were transferred into syngeneic nude B6 recipients subsequently engrafted with two bm12 skin grafts. These CD4^+^ T-cells express a transgenic TCR that specifically recognizes the MHC class II molecule I-A^bm12^ found on B6.C-H2bm12 (bm12) mice. This model allows us to track alloantigen specific CD4^+^ T-cells and yield specific information about an age-dependent T-cell migration, differentiation and activation pattern.

**Figure 7:**
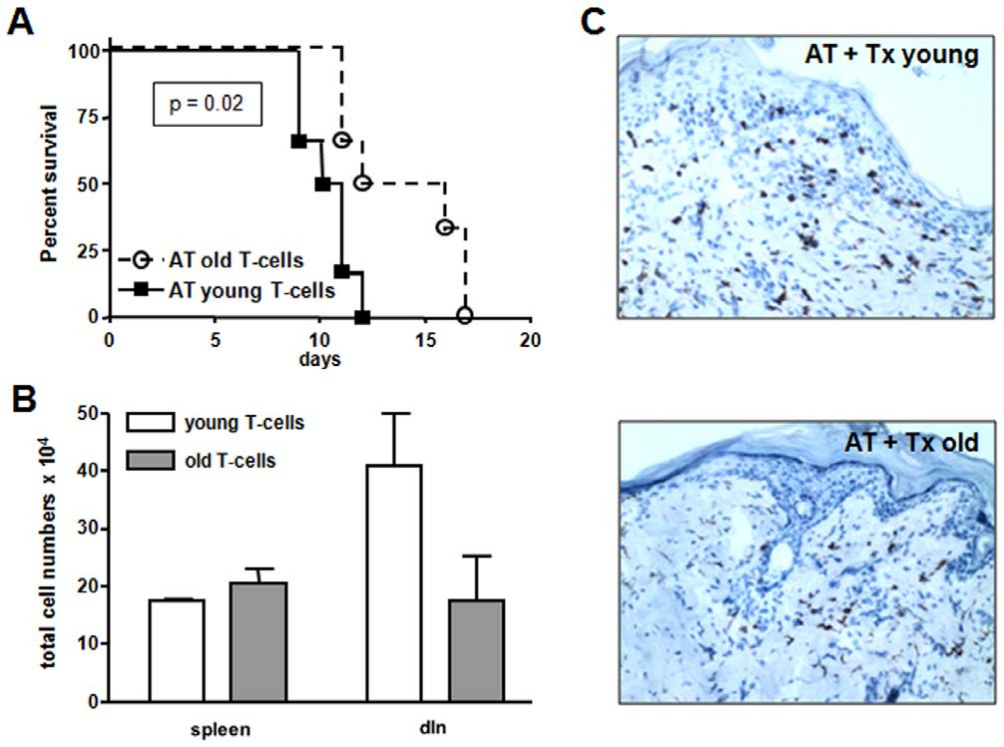
Impaired CD4^+^ T-cell expansion leads to prolonged graft survival. Impaired proliferation of alloantigen-specific CD4^+^ T-cells leads to prolonged graft survival in old skin graft recipients. Nude B6 mice were engrafted with two bm12 skin transplants following adoptive transfer of young or old 2×10^6^ transgenic CD4^+^ ABM T-cells. In line with the reduced CD4^+^ T-cell response in old WT recipients skin graft survival was significantly prolonged following transfer of old alloreactive CD4^+^ T-cells (**A**). One week after transplantation spleen, dLN and ndLN were harvested and leukocytes were stained for CD4^+^, Vα2^+^ and Vβ8^+^ expression to assess proliferation of adoptively transferred T-cells (**B**). In parallel, skin grafts were stained for graft infiltrating CD4^+^ T-cells (**C**). At day 7, absolute numbers of adoptively transferred CD4^+^ T-cells were counted in different lymphatic compartments in order to determine T-cell proliferation. A significantly reduced proliferation of old tg CD4^+^ T-cells was accompanied by decreased graft infiltration, underscoring a delayed rejection response (**B** and **C**).

FACS analysis of the purified CD4^+^ T-cells prior to the adoptive transfer showed a higher frequency of CD4^+^CD44^high^CD62L^low^ T-cells in old transgenic animals. Frequencies of activated T-cells (CD4^+^CD25^+^ and CD4^+^CD69^+^, respectively, data not shown) were not different between young and old purified CD4^+^ T-cells. However, following the transfer of 2×10^6^ ABM CD4^+^T-cells, recipients of old T-cells showed a significantly delayed graft rejection compared to recipients of young T-cells (MST: 14.0 days vs. 10.5 days, p<0.02, **Fig. 7**). This data suggest that delayed skin graft rejection in older WT recipients is associated with an age-dependent impaired allospecific T-cell response.

### Skin Graft Infiltration by Adoptively Transferred CD4^+^T-Cells

Immunohistochemical staining of skin grafts harvested on post-operative day 7 revealed fewer infiltrating CD4^+^ T-cells in recipients of old ABM tg T-cells than in grafts harvested from recipients of young T-cells (**Fig. 7**). While characteristics of skin graft infiltration were consistent with the impaired CD4+ T-cell proliferation and delayed skin graft rejection by adoptively transferred old T-cells, young and old WT recipients had shown a comparable graft infiltration. This discrepancy may be explained by an impaired proliferation of old memory T-cells in “empty” hosts as underlined by fewer effector/memory T-cells in draining lymph nodes.

### Impaired Allospecific T Cell Expansion and Activation after Adoptive Transfer of Old CD4^+^ T-Cells

**Figure 8:**
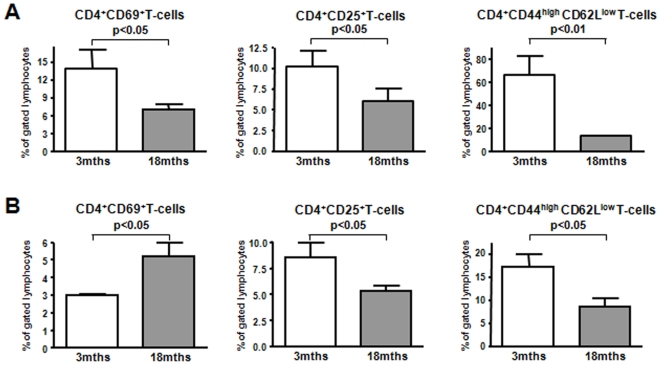
Reduced proliferation of old alloreactive effector/memory CD4^+^ T-cells. Old alloreactive effector/memory CD4^+^ T-cells proliferate less in response to antigen. Following adoptive transfer of young or old 2×10^6^ transgenic CD4^+^ ABM T-cells nude B6 mice were engrafted with two bm12 skin transplants. One week later spleen (**A**) and dLN (**B**) were retrieved, leukocytes were stained for CD4, Vα2 and Vβ8 expression and the expression of different cell surface markers (n = 4). Relative numbers of CD69^+^ and CD25^+^CD4^+^ T-cells were reduced in recipients spleens after transfer of old alloreactive T-cells indicating an impaired activation and proliferation. Similarly, alloreactive effector/memory CD4^+^ T-cells were significantly reduced 7 days after restimulation in vivo (**A**). In contrast, enhanced early activation (CD69^+^) of old tg CD4^+^T-cells in draining lymph nodes did not lead to a stronger expansion of effector/memory T-cells most likely due to an impaired IL-2 production (**B**).

Adoptively transferred old T-cells proliferated less in response to their specific antigen. Likewise, percentages of very early activated CD4^+^CD69^+^ T-cells were decreased in the spleen. While purified old ABM T-cells had shown a higher frequency of effector/memory T-cells prior to transfer, 7 days after in vivo re-stimulation a lower percentage of CD4^+^CD44^high^CD62L^low^ T-cells was detected (**Fig. 8**). These findings are in contrast to an enlarged memory T-cell pool in WT recipients suggesting further impairment of allospecific memory T-cells with increasing age [8], [10].

Furthermore, intracellular cytokine staining revealed a different cytokine profile of old and young alloreactive T-cells in dLN which was comparable to the cytokine expression in WT recipients (data not shown).

In summary, an overall impaired expansion of aged alloreactive CD4^+^ T-cells was observed. The majority of transferred old CD4^+^ T-cells did not proliferate after transplantation even though activation seemed to be unimpaired as indicated by increased CD69 and IFNγ expression. In particular, old alloreactive effector/memory T-cells may be unresponsive as frequencies significantly decreased after antigen encounter.

## Discussion

Immunological changes associated with increasing recipient age have become an important challenge in clinical transplantation, yet information on an age-associated immune response after organ transplantation remains scarce. As alterations of the T-cell response with age may require specific adjustment of immunosuppression, more detailed investigation on the adaptive immune response in old recipients will have important clinical implications. The aim of our current study was to investigate CD4^+^ T-cell activation and differentiation in old recipients. In accordance with previously reported age-related changes of the CD4^+^ T-cell pool, higher frequencies of early-activated T-cells and effector/memory T-cells were found in spleens of naive old mice. However, these phenotypical changes did not correspond with functional properties as intracellular cytokine production by CD4^+^T-cells was not enhanced. Moreover, splenocyte proliferation and alloreactive IFNγ production in vitro were significantly impaired in naive old animals.

In accordance with these findings, rejection of MHC Class II mismatched skin grafts was significantly delayed in aged wild-type recipients. Despite more pronounced activation characteristics, old recipients were unable to mount as strong an immune response as young animals. This finding was further supported by a diminished proliferation and cytokine production in vitro.

In keeping with previously published results decreased frequencies of IL-2^+^ T-cells were detected in aged WT recipients causing a reduced proliferative response [7], [8], [17]. Interestingly, findings in draining lymph nodes were suggestive of a delayed T-cell activation as frequencies of CD25^+^ and CD69^+^ T-cells were diminished.

While an increased percentage of IFNγ^+^ T-cells, but also IL-10^+^ T-cells, was detected in spleens of old WT recipients, functional properties were significantly impaired. In the presence of a higher percentage of aged effector/memory T-cells this finding may be indicative of a decreased function of old memory T-cells as described by Haynes et al. [7].

In line with our own ELISPOT findings, Tesar et al. also reported on reduced IFNγ production of aged CD4^+^ memory T-cells after transplantation [10]. Using a fully mismatched skin transplant model, they showed that a lack of IL-2 production was associated with an increased donor-specific Th17 response in aged transplant recipients leading to a delayed rejection response. As we also observed significantly fewer IL-2 expressing CD4^+^ T-cells in the aged arm, it seems conceivable that the impaired Th1 response observed in our model may in part be compensated for by an enhanced IL-17 production.

Alloantigen-specific T-cells constitute a small percentage among all T-cells. As aging presumably leads to accumulation of alloantigen-specific T-cells, we sought to further dissect the CD4^+^ T-cell response. We aimed to analyze the subset of alloantigen-specific T-cells which may compensate for a reduced functionality of the majority of old memory T-cells. For this purpose, a well-established transgenic model was employed that allowed us to overcome the low precursor frequency of alloreactive T-cells in wild-type animals and to track the alloantigen specific CD4^+^ subpopulation [12]. Comparable to the results obtained in wild-type mice, old alloantigen-specific tg TCR^+^ CD4^+^T-cells showed a significantly delayed graft rejection.

While the percentage of aged effector/memory T-cells had increased prior to adoptive transfer (AT), a lower frequency was found in the spleen after AT indicating an impaired expansion and generation of new effector T-cells.

According to the overall reduced expansion of the transferred T-cells, frequencies of CD25^+^ T-cells and IL-2^+^ T-cells were decreased as compared to young controls. Furthermore, a higher percentage of CD69^+^ T-cells indicated a preserved activation of old alloreactive T-cells. However, the significantly impaired proliferation of old T-cells prevented further differentiation and acquisition of an effector/memory phenotype.

We detected similar frequencies of CD4^+^CD25^+^FoxP3^+^ regulatory T-cells in naive young and old mice. In contrast, one week after transplantation regulatory T-cells had significantly expanded in spleens of old recipients paralleling the enhanced numbers of activated and effector/memory T-cells. Our results are consistent with other reports on increased numbers of CD4^+^CD25^+^FoxP3^+^ T-cells in aging [18]–[21].

Most previous studies on regulatory T-cells function have not demonstrated a significant age-associated functional decline [20], [21]. Similarly we demonstrated that old CD4^+^CD25^+^FoxP3^+^ regulatory T-cells suppressed the proliferation of co-cultured young responder T-cells as sufficiently as young regulatory T-cells. Our findings suggest that the balance of effector and regulatory mechanisms is skewed towards a suppressed immune response in the elderly.

While our current study focused on the role of CD4^+^ T-cells and the function of regulatory T-cells in elderly recipients, the importance of age-dependent changes of CD8^+^ T-cells is well-recognized. Studies describing the aging immune response have demonstrated that aging of the CD8^+^ T-cell compartment is associated with the oligoclonal expansion of a memory T-cell subpopulation with reduced CD28 expression and impaired intracellular signaling. Hence, antiviral immunity is impaired in the elderly [22], [23]. As the frequency of naive CD8^+^ T-cells seems to be less affected by thymic involution, a reduced CD4^+^/CD8^+^ ratio contributes to an altered age-dependent immune response. In a previous study, we were able to demonstrate an impaired CD4^+^ but elevated CD8^+^ T-cell count, which was associated with long term graft deterioration [5]. A recent study demonstrated prolonged survival of fully mismatched skin allografts in recipients of old T-cells associated with an impaired expansion of transplant reactive CD8+ T cells [24].

In conclusion, we have shown a muted response to antigen in naive old mice. The accumulation of memory T-cells with a diminished activation potential lead to a hyporesponsive T-cell pool. A significantly decreased proliferation of aged CD4^+^ T-cells caused by an impaired IL-2 production contributed to a delayed rejection with reduced graft infiltration. Besides, the well-preserved regulatory T-cell function contributed to the observed ameliorated alloimmune response in old recipients. These results may have important implications for an age-dependent immunosuppressive therapy.
